# The three-body problem of therapy with induced pluripotent stem cells

**DOI:** 10.1186/s13073-015-0141-7

**Published:** 2015-02-20

**Authors:** Jakub Tolar, John A McGrath

**Affiliations:** Stem Cell Institute and Division of Blood and Marrow Transplantation, Department of Pediatrics, University of Minnesota, 420 Delaware St SE, MMC 366, Minneapolis, MN 55455 USA; St John’s Institute of Dermatology, King’s College London, Floor 9 Tower Wing, Guy’s Hospital, Great Maze Pond, London, SE1 9RT UK

## Abstract

Regenerative medicine has a three-body problem: alignment of the dynamics of the genome, stem cell and patient. Focusing on the rare inherited fragile skin disorder epidermolysis bullosa, three recent innovative studies have used induced pluripotent stem cells and gene correction, revertant mosaicism or genome editing to advance the prospects of better cell-based therapeutics to restore skin structure and function for epidermolysis bullosa and potentially other inherited diseases.

One of the dominant ambitions of medicine today is for genes and cells to be used as medications. However, cells and genes do not operate independently of their environment, but always in the context of the recipient. The default of cellular transplantation is rejection, the innate and adaptive immune system protecting the host body. We can apply the key concepts of transplantation biology, tested over 50 years of bone marrow transplants, to the development of graftable induced pluripotent stem cell (iPSC)-derived cells and tissues. Three recent publications [1-3] extend iPSC-based therapy initiatives in the field of regenerative dermatology and exemplify a larger challenge for any clinically meaningful medical approach: the need to simultaneously engineer genes, capture cellular stemness and graft gene-corrected cells into individuals with an inherited skin disease.

## A shipwreck, not a butterfly

In the severe forms of epidermolysis bullosa (EB), a group of skin fragility disorders with profound implications for physical and mental health, even slight friction causes the layers of mucocutaneous membranes to slide apart and results in painful wounds that can resemble severe burns. The most overwhelming of these skin conditions are recessive dystrophic EB (RDEB) and junctional EB (JEB), autosomal recessive disorders in which the genes encoding major skin adhesion proteins do not function properly, leading to severely diminished or absent gene expression. Patients with these disorders are often called ‘butterfly children’ because of their delicate and easily damaged skin, and the fact that many do not survive into adulthood.

This disorder has an impact far beyond the skin, as these individuals experience severe skin blistering, corneal erosions, and mucosal wounds that can result in malnutrition. EB is a horrible and frequently fatal disease that wrecks any attempt at a normal life, both for the sufferer and for the family. Despite the intense efforts of medical scientists around the globe, there is currently no cure. However, as the work from three teams discussed herein demonstrates [1-3], scientists are working with determination and creativity on a cure.

## Furry test tubes

Murine models have proven tremendously useful in studying the basic biology of human inherited skin diseases and in preclinical modeling of potential therapeutic interventions. For RDEB, at least two murine models exist, one with no expression of basement membrane type VII collagen (C7) [4], and one with approximately 10% wild-type expression of C7 [5]. The groups of Penninger and Bruckner-Tuderman [1] used the latter model, and reprogrammed tail skin fibroblasts into iPSCs that were used for therapy. To demonstrate the feasibility of this iPSC-based therapy, mutant cells were corrected, restoring the function of *Col7a1*. These corrected iPSCs were then differentiated back into fibroblasts, and injected intradermally into mutant mice. Expression of C7 increased over the first 8 weeks and then declined to the baseline levels expected in this model of RDEB (corresponding with the decline of donor cells to undetectable numbers over the same period). Importantly, no obvious abnormal inflammatory response, fibrosis or tumor formation (such as teratoma derived from an errant iPSC, or squamous cell carcinoma (SCC) associated with the pathophysiology of RDEB) was observed over the 18 weeks after therapy. To demonstrate functionality of the new C7, the authors tested the skin stability and observed it increased after injection of corrected fibroblasts, but not after administration of uncorrected mutant cells. This is a key observation, since previous work indicated that injection of cells or just a cell-free solution can also increase expression of mutant C7 at the epidermal-dermal junction in human RDEB subjects with hypomorphic *COL7A1* mutations [6] and improve wound healing, presumably in part by changing a chronic wound into an acute one.

## Rebooted skin cells

Equally important to advances in the clinical application of iPSC therapy has been reprogramming keratinocytes, the major cell type expressing C7 in normal skin, with genetic reversion of the disease-causing mutations. Revertant mosaicism occurs in some RDEB patients, providing a source of naturally gene-corrected skin cells. Researchers have previously generated personalized iPSCs and iPSC-derived skin cells from individuals with JEB [7], RDEB [8] and mosaic RDEB [9]. Now Christiano and colleagues [2] have used keratinocytes with a naturally occurring reversion in the *COL17A1* gene (encoding type XVII collagen) from a healthy appearing skin patch of an individual with JEB, reprogrammed them into iPSCs, and differentiated them into keratinocytes with the capacity to form skin-like organoids. These advances are elegant and promising tools in future EB therapies, even though three key challenges remain: the iPSCs were generated with retroviral-mediated transgenesis, which is unlikely to be acceptable in clinical trials; the skin-like equivalents are not true skin grafts; and the graftable keratinocytes have not yet been tested in a murine model of EB.

## Have a nice DNA

The brilliant approach represented by the work of Oro and colleagues [3] is aimed at helping the majority of people with EB who do not have clinically identifiable mosaic cells (or in whom the gene reversion leads to only partial restoration of collagen expression). They propose gene correction of EB-causing mutations by gene editing. In contrast to the predominant current approach to gene therapy using viral vectors to deliver the gene of interest to the genome (gene addition), gene editing employs homology-driven repair to replace the disease-causing mutation *in situ*, in principle leaving no other footprint in the genome. This is important, as the viral vectors used in the past led in several instances to position effect after genomic insertion, activation of proto-oncogenes, and cancer. Gene editing has been used previously for correction of human RDEB mutation in skin cells reprogrammed to iPSCs and differentiated to keratinocytes [10], but in this work they used highly recombinogenic adeno-associated virus to mediate *COL7A1* gene editing in RDEB fibroblasts, which they could reprogram to pluripotency and differentiate into keratinocytes. They have performed a thorough analysis of expression profiles and off-target effects in gene-edited cells and found no alterations with identifiable genotoxicity.

## Second skin is not enough

Many new questions arise as the boundaries of genome and cellular engineering are explored. Firstly, it remains to be determined whether iPSC-derived skin cells can be induced to maintain long-lasting functionality, whether they can be injected repeatedly without side effects, and whether the extracellular matrix of EB skin can be modified to improve their therapeutic benefit. Secondly, the corrective mechanisms underpinning revertant mosaicism are not yet clear. How many cells are needed for clinically meaningful change in skin stability, and what is the *in vivo* selection advantage, if any, of the reverted cells? Finally, wounds in EB arise both inside and outside the body, in varying severity and longevity, and at any time. Using skin grafting for all the wounds may not be feasible, especially given that the skin is the largest organ of the body. Another difficulty is that EB is a systemic disease with not just cutaneous wounds, but also mucosal wounds (esophagus and mouth), corneal abrasions, and other organ malfunctions (such as chronic anemia, kidney dysfunction and bone/dental disease). The risk of SCC, typically aggressive, metastatic and lethal in severe generalized forms of EB, will also likely persist as long as any wounds remain.

Thus, the future of iPSC-based therapies in regenerative dermatology is difficult to predict. The customized gene-corrected cell is as yet only a tool. To realize its potential clinical benefit, it must be transplanted into the right environment and accepted, at least transiently. Thus, although none of these three tasks, collagen gene correction, skin cell culture and transplantation into individuals with EB, are singly sufficient, all are necessary as researchers continue to pursue ways to relieve the effects of this disorder. In the future, these three separate scientific foci must align and integrate to successfully see translation of exciting science into the clinic. This same approach is needed to grow confidence in applying iPSCs to other genetic disorders and to regenerative therapies in general.

## Abbreviations

C7: Type VII collagen
EB: Epidermolysis bullosa
iPSC: Induced pluripotent stem cell
JEB: Junctional epidermolysis bullosa
RDEB: Recessive dystrophic epidermolysis bullosa
SCC: Squamous cell carcinoma
